# Are MUPs a Toxic Waste Disposal System?

**DOI:** 10.1371/journal.pone.0151474

**Published:** 2016-03-11

**Authors:** Jae Kwak, Eva Strasser, Ken Luzynski, Michaela Thoß, Dustin J. Penn

**Affiliations:** 1 Research Institute of Wildlife Ecology, Department of Integrative Biology and Evolution, University of Veterinary Medicine Vienna, Vienna, Austria; 2 Konrad Lorenz Institute of Ethology, Department of Integrative Biology and Evolution, University of Veterinary Medicine Vienna, Vienna, Austria; Duke University, UNITED STATES

## Abstract

Male house mice produce large quantities of major urinary proteins (MUPs), which function to bind and transport volatile pheromones, though they may also function as scavengers that bind and excrete toxic compounds (‘toxic waste hypothesis’). In this study, we demonstrate the presence of an industrial chemical, 2,4-di-tert-butylphenol (DTBP), in the urine of wild-derived house mice *(Mus musculus musculus)*. Addition of guanidine hydrochloride to male and female urine resulted in an increased release of DTBP. This increase was only observed in the high molecular weight fractions (HMWF; > 3 kDa) separated from male or female urine, suggesting that the increased release of DTBP was likely due to the denaturation of MUPs and the subsequent release of MUP-bound DTBP. Furthermore, when DTBP was added to a HMWF isolated from male urine, an increase in 2-*sec*-butyl-4,5-dihydrothiazole (SBT), the major ligand of MUPs and a male-specific pheromone, was observed, indicating that DTBP was bound to MUPs and displaced SBT. These results suggest that DTBP is a MUP ligand. Moreover, we found evidence for competitive ligand binding between DTBP and SBT, suggesting that males potentially face a tradeoff between eliminating toxic wastes versus transporting pheromones. Our findings support the hypothesis that MUPs bind and eliminate toxic wastes, which may provide the most important fitness benefits of excreting large quantities of these proteins.

## Introduction

Major urinary proteins (MUPs) are members of the lipocalin family that can sequester and transport a variety of lipophilic molecules in blood and other hydrophilic body fluids [1]. Male house mice produce large quantities of MUPs (20–40 mg of protein per day [2]), which function to bind and transport volatile pheromones to scent marks and stabilize their release [3, 4]. MUPs have been suggested to have a potentially more important function by acting as scavengers that bind and excrete toxic compounds. This ‘toxic waste hypothesis’ has been independently suggested by two different laboratories [5, 6]. It is consistent with the detoxification function of other lipocalins [7] and would help explain why *Mup* genes are expressed in the liver.

Previous studies showed that the major portions of xenobiotics (defined as ‘administered drugs or environmental contaminants’) that are excreted in mouse urine are bound to MUPs. Larsen et al. [8] found that when a radiolabeled methylsulphonyl metabolite of a polychlorinated biphenyl (PCB; a banned industrial chemical that may act as a carcinogen and/or an endocrine disruptor) was administered intraperitoneally to male mice, significant radioactivity was excreted in mouse urine and associated with MUPs. Robertson et al. [9] observed that after a subcutaneous injection of menadione (a synthetic chemical added to commercial mouse food as a nutritional supplement), this chemical was bound to MUPs when excreted in urine. Recently, Hakk et al. [10] demonstrated the excretion of radiolabeled 2,3,7,8-tetrachlorodibenzo-*p*-dioxin (TCDD; a carcinogenic environmental contaminant) in urine and its binding to MUPs when it was administered orally to male mice. The excretion of xenobiotics in the form of a MUP-ligand complex is also observed in female mice. Staskal et al. [11] reported that only one major compound corresponding to the parent chemical was detected in the urine samples collected from female mice after oral administration of 2,2’,4,4’-tetrabromodiphenyl ether (BDE47; a banned industrial chemical with harmful effects on the liver, thyroid, and neurobehavioral development in animals). Staskal et al. [12] later confirmed that the majority of BDE47 excreted in female urine was bound to MUPs. Kwak et al. [6] revealed that butylated hydroxytoluene (BHT), an antioxidant present in mouse diet, was excreted and bound to MUPs in female mouse urine. These studies suggest that toxic and potentially toxic xenobiotics can be removed with the aid of MUPs and that MUPs potentially function as a defense mechanism by binding and eliminating toxic waste in mice. Previous studies have only examined this hypothesis in inbred, laboratory mice and therefore studies are needed to investigate this hypothesis in outbred or wild mice, especially living in more natural ecological conditions.

We took the opportunity to investigate the toxic waste hypothesis during a recent study on the regulation of MUPs in wild-derived mice (F1 offspring of wild-caught *Mus musculus musculus)*. In all urine samples analyzed, we unexpectedly detected 2,4-di-tert-butylphenol (DTBP). DTBP is an industrial chemical mainly used as an intermediate precursor for producing synthetic antioxidants [13]. It also exhibits a strong antioxidant activity [14]. Although it was detected in the mouse diet used in the study, it is likely to be a contaminant which may be in contact with the diet during the manufacturing or packaging process since it is not intended to be used as an ingredient or additive in its own right [15]. To understand the binding interaction of MUPs and the elimination of xenobiotics, we investigated whether the exogenous compound is bound to MUPs and excreted in urine samples collected from male and female mice. We aimed to determine whether MUPs function to eliminate toxic waste since such a mechanism has potentially more important fitness benefits than pheromone transport. Finally, we suggest that if competitive ligand binding occurs, then males potentially face tradeoffs between eliminating toxic wastes versus pheromone signaling due to competition for MUP binding pockets.

## Materials and Methods

### Chemicals

2,4-Di-tert-butylphenol (DTBP; product # 137731) and guanidine hydrochloride (GdmCl; product # G3272) were purchased from Sigma-Aldrich (Vienna, Austria). 2-*sec*-Butyl-4,5-dihydrothiazole (SBT) is not commercially available, but its identity was previously confirmed with a synthesized chemical [6].

### Animals and standard housing

Experimental animals were F1 offspring of wild-caught house mice (*Mus musculus musculus*) trapped at seven locations within a 300 m radius in Vienna (48°13’14” N; 16°17’00” E). The F1 mice were weaned at the age of 21 ± 1 days, housed in sibling groups until the age of 35 ± 1 days and subsequently housed individually in standard mouse cages (type IIL, 36.5 x 20.5 x 14 cm, product # 1284L001, Tecniplast, Germany) containing wooden bedding (product # LTE E-002, ABEDD, Austria), a cardboard toilet paper roll, two cotton Nestlets (product # 3097055, Ehret, Austria), and a red house (product #ACRE011, Tecniplast, Germany). Food (rodent diet, product # 1324, Altromin, Germany) and water were provided *ad libitum* and temperature was maintained at 22 ± 2°C. Mice were kept on a 12:12 h light:dark cycle with red light on at 1500. At the start of the experiment, animals were three to six months old.

### Semi-natural housing conditions

As part of a larger experiment, 128 individual mice were assigned to one of two treatment groups: enclosure group or caged control group. For three months, enclosure mice (N = 64) lived in large (3.4 x 4 m each) seminatural enclosures containing wooden bedding (product # LTE E-002, ABEDD, Austria), plastic nest boxes, a water station, wood wool, and paper towels as nesting material. Food (rodent diet, product # 1324, Altromin, Germany) and water were provided *ad libitum*; temperature was maintained at 22 ± 2°C and the mice were kept on a 12:12 h light:dark cycle with red light on at 1500. The caged controls (N = 64) were litter mates of the enclosure group and kept under standard housing conditions (see above).

### Urine collection

Urine collections were conducted under red light conditions at the beginning of the dark cycle. Metabolic cages (product # 3600M021, Techniplast, Germany) were used for 1h urine collection and urine samples were transferred to glass vials and stored at -80°C.

### Collection of DTBP and 2-sec-butyl-4,5-dihydrothiazole released from mouse urine

Fifteen microliters of intact or denatured urine were placed in a 4 mL glass vial and a 2 cm, three-component solid phase microextraction (SPME) fiber (30 μm carboxen, 50 μm divinylbenzene, polydimethylsiloxane; Supelco Corp., Bellefonte, PA, USA) was used for collection of the headspace DTBP and SBT released from urine in the vial. The vial was submerged in a water bath at 37°C and was equilibrated for 10–15 min. Then, the headspace containing these compounds was extracted by the SPME fiber for 15 min at 37°C. The urine sample in the vial was agitated using a magnetic stirrer during the equilibration period, but not in the extraction period. The SPME fiber containing the adsorbed compounds was then inserted into the injection port of a gas chromatograph–mass spectrometer (GC–MS) and desorbed for 1 min at 240°C.

### Fractionation of urine by centrifugal filtration

One hundred fifty microliters of each male and female pooled urine sample were placed on a Vivaspin 500 3kDa molecular weight (MW) cutoff (GE Healthcare, Little Chalfont, UK) and spun at 15 000 g for 30 min at room temperature. We obtained two fractions: MW < 3 kDa and MW > 3 kDa. Each fraction was extracted by SPME with or without addition of GdmCl followed by GC-MS analysis.

### Analysis of DTBP and SBT by gas chromatography–mass spectrometry

A Shimadzu GC–MS QP2010 Plus (Duisburg, Germany) was used for separation and analysis of these compounds. Two different GC columns were used in this study. A HP-5ms column (30 m × 0.25 mm with 0.25 μm film thickness; Agilent, Vienna, Austria) and a Supelcowax 10 column (30 m × 0.25 mm with 0.50 μm film thickness; Sigma-Aldrich, Vienna, Austria) were used for the analyses of the urine samples and the fractionated urine samples, respectively. The Supelcowax column was installed for a different study after the urine sample analyses, and the analyses of the fractionated urine samples were subsequently performed on the column. Nevertheless, the use of different columns did not influence the detection of DTBP and SBT in the samples analyzed. The GC oven temperature for the analysis with the HP-5ms column was programmed at 8°C/min from 60 to 230°C with a 1.25-min hold at the final temperature. For the analysis with the Supelcowax column, the oven temperature was held at 40°C for 1 min, then programmed at 6°C/min to 220°C with a 9-min hold at this final temperature. Helium was used as the carrier gas at the linear velocity of 38.9 cm/sec. The injection port was held at 240°C. The transfer line temperature between GC and MS was 250°C. Operating parameters for the mass spectrometer were as follows: ion source temperature at 200°C; electron impact ionization (70eV); and scanning frequency was 4/s from m/z 41 to m/z 300.

### Monitoring the release of DTBP from denatured urine

GdmCl, a protein denaturant, was added to intact urine to determine whether DTBP is a MUP ligand. Previous studies revealed that VOCs whose headspace concentration increased upon denaturation were ligands released from urinary proteins [6, 16, 17]. The denaturation was accomplished by adding 20 mg GdmCl into a vial containing 15 μL of intact urine. The total concentration of GdmCl in urine was approximately 8M. Each urine sample was allowed to be denatured for an hour at room temperature prior to collection of DTBP released from the sample by SPME.

### Investigation of “salting out” effect of GdmCl on DTBP

A DTBP stock solution (1 mg/mL in deionized water) was prepared. The majority of DTBP was not dissolved since it is generally not soluble in water. The dissolved portion of DTBP was further diluted with deionized water (1:25). Two hundred microliters of the diluted DTBP solution were added to a 4 mL glass vial, and the headspace DTBP released from the vial was analyzed in the presence or absence of 150 mg GdmCl. DTBP was collected by SPME and analyzed by GC–MS as mentioned above.

### Displacement of SBT by DTBP

Two hundred micrograms of DTBP and the high molecular weight fraction obtained by centrifugal filtration from male urine were placed into a 4mL glass vial, and allowed to be mixed for three hours at room temperature. Then, SBT released into the headspace from the sample was analyzed.

## Results

**Fig 1:**
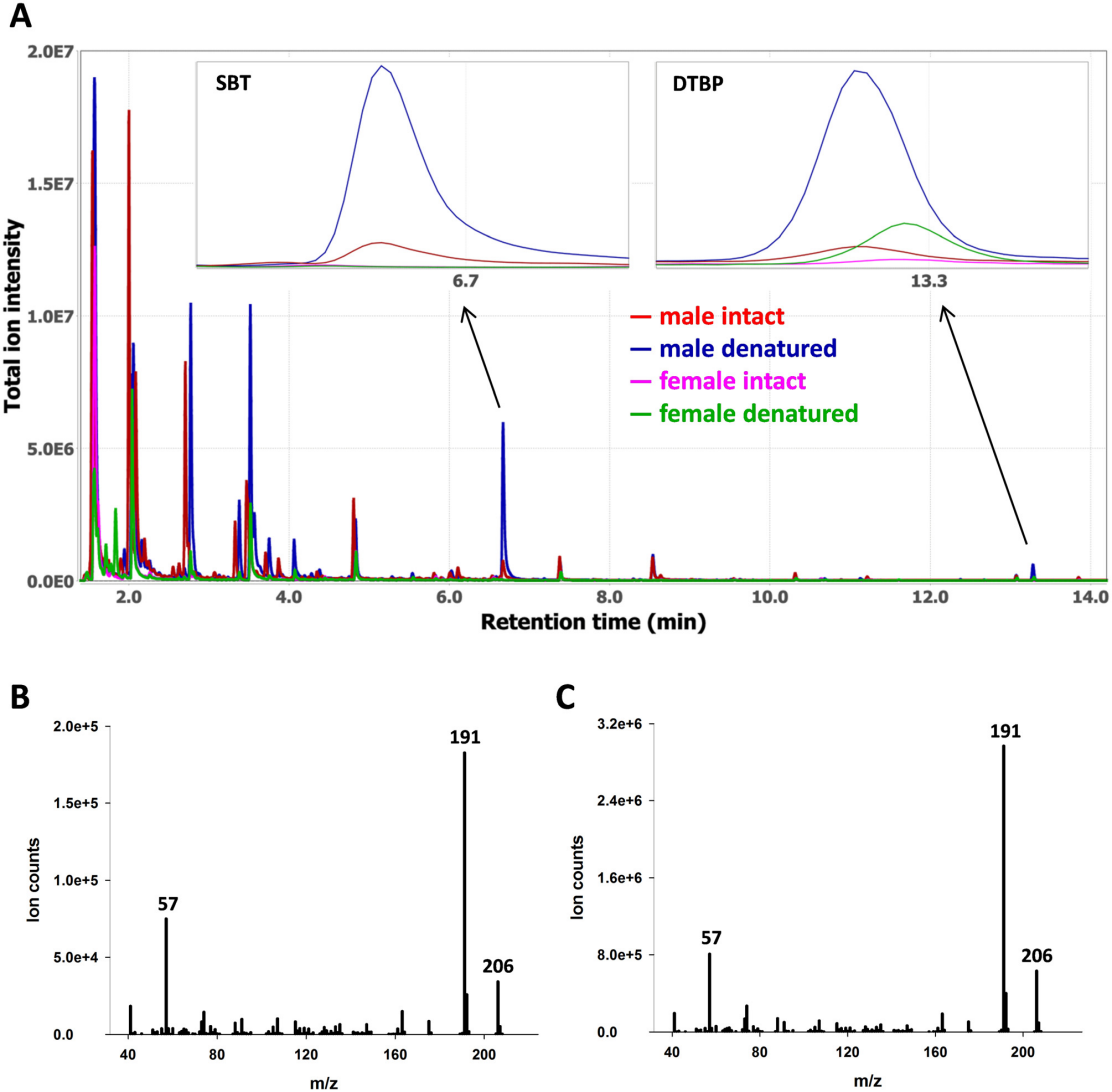
Representative overlaid chromatograms acquired from intact and denatured male and female mouse urine samples (A), the mass spectra of DTBP obtained from a male mouse urine sample (B) and a synthetic standard (C). The intensity distributions of DTBP and SBT in the urine samples are shown in the insets (A). Guanidine hydrochloride (GdmCl) was added to urine to denature urinary proteins.

**Fig 2:**
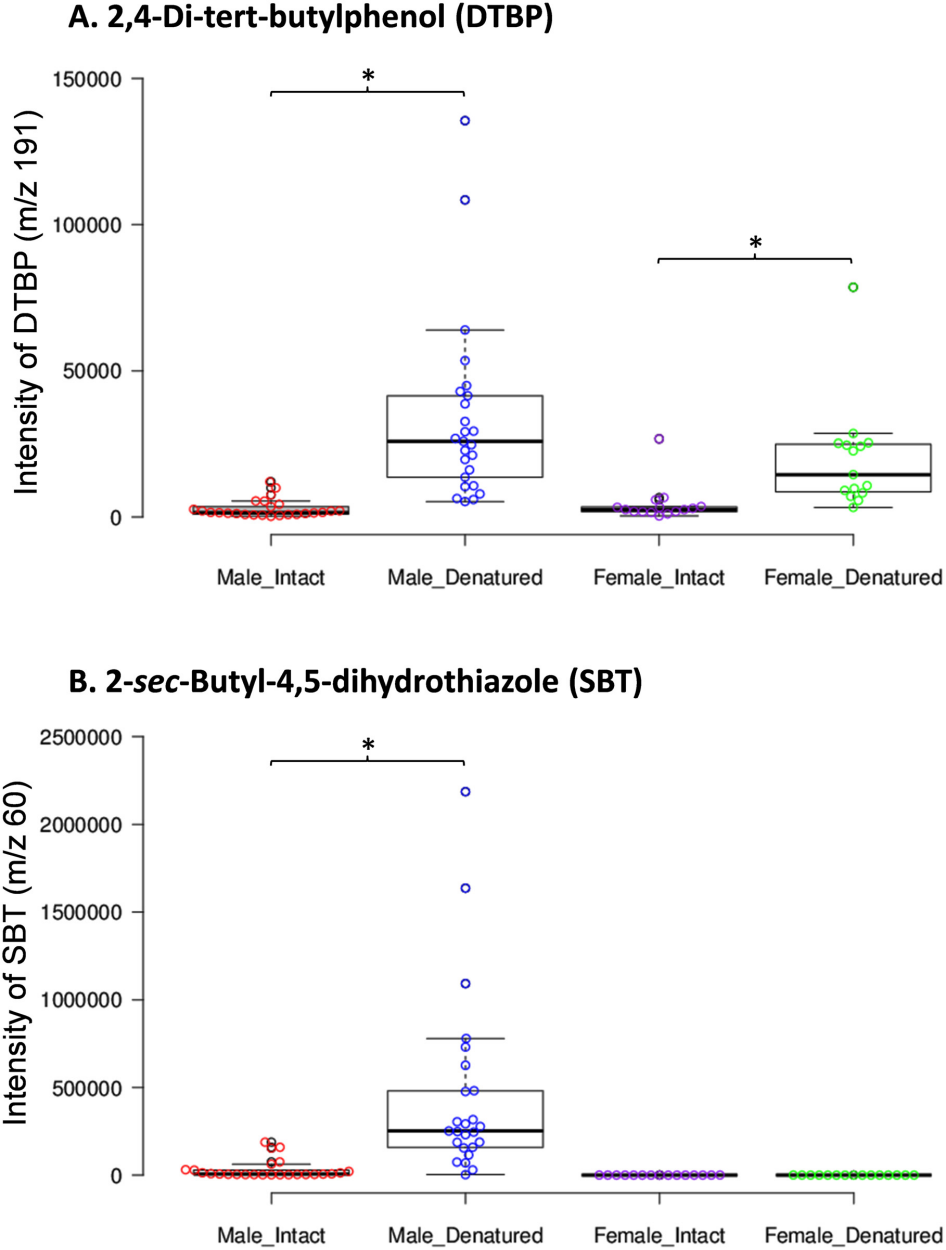
The release of DTBP (A) and SBT (B) upon denaturation of male and female mouse urine samples by addition of GdmCl. The box plots were created online using the BoxPlotR application [18]; http://boxplot.tyerslab.com/ *P ≤ 0.01 (Mann–Whitney test).

A total of 40 mouse urine samples (25 male and 15 female) were analyzed in both intact and denatured conditions, and Fig 1A shows representative overlaid chromatograms from these samples. The retention time and mass spectrum of DTBP detected in the samples matched those of a synthetic standard, and the mass spectra of DTBP obtained from a male mouse urine sample versus a synthetic standard are shown in Fig 1B and 1C, respectively. The increased release of DTBP was observed upon addition of GdmCl, a protein denaturant, to male or female urine samples (Fig 1A right inset and Fig 2A). The levels of DTBP were significantly different between intact and denatured samples collected from male (U = 15.0, p < 0.01) or female mice (U = 20.0, p < 0.01). The increased release of SBT, a male-specific MUP ligand, was also observed in male samples (Fig 1A left inset and Fig 2B), as reported previously [6], and SBT levels significantly increased with addition of GdmCl (U = 43.0, p < 0.01). The increased release of SBT is due its dissociation from denatured MUPs, confirming that SBT is a MUP ligand [6, 16, 17]. Similarly, the increased release of DTBP is likely due to the denaturation of MUPs and its subsequent dissociation from MUPs.

**Fig 3:**
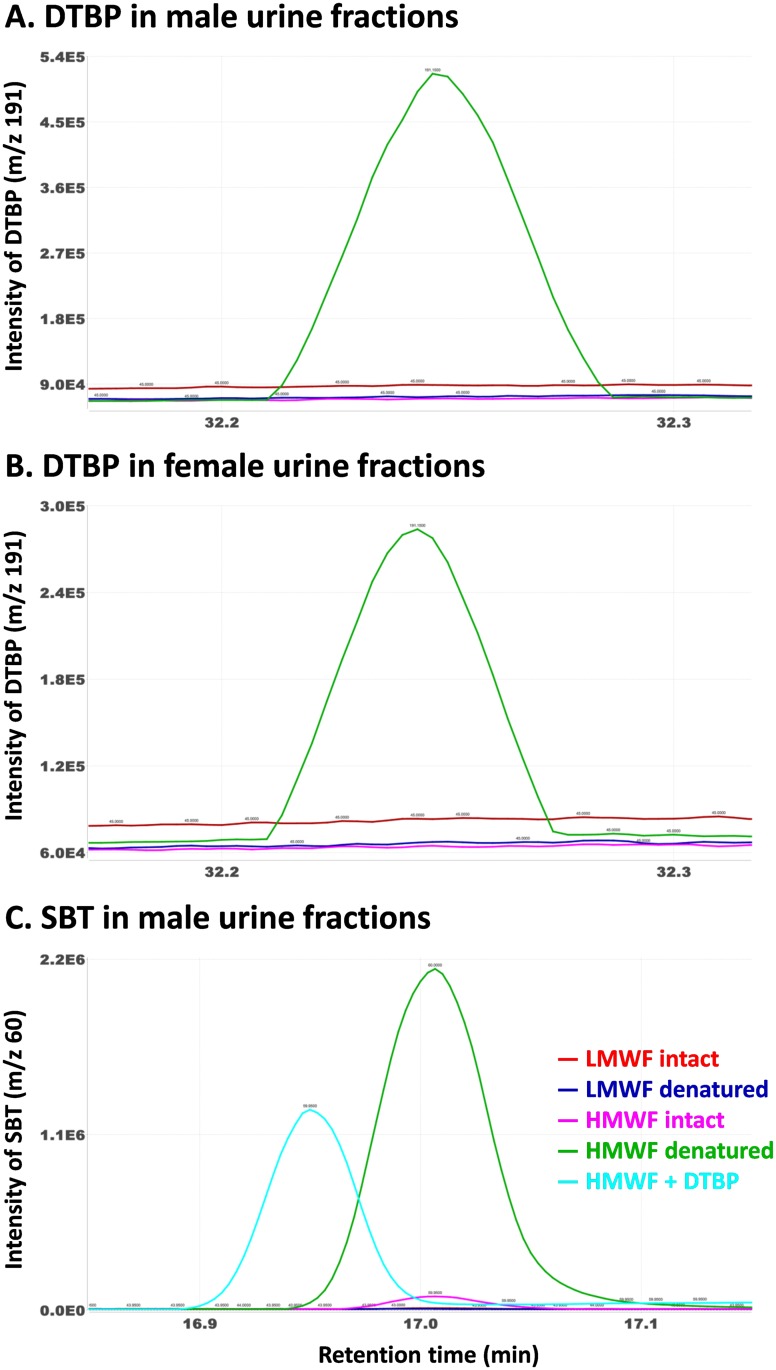
The distribution of DTBP in the low molecular weight fraction (LMWF; MW < 3 kDa) and high molecular weight fraction (HMWF; MW > 3 kDa) obtained from male (A) and female (B) urine, that of SBT in the male urine fractions, and the displacement of SBT by DTBP (C).

In order to measure the distribution of unbound and bound DTBP and SBT to proteins in urine, male and female urine samples were fractionated by centrifugal filtration and two fractions were obtained: low molecular weight fraction (LMWF; MW < 3 kDa) and high molecular weight fraction (HMWF; MW > 3 kDa). As shown in Fig 3A and 3B, DTBP was exclusively associated with the HMWFs isolated from both male and female urine and released after addition of GdmCl. Furthermore, the majority of SBT was associated with the HMWF obtained from male urine (Fig 3C). These results strongly suggest that DTBP and SBT are largely bound to urinary proteins (≥ 95% of which are MUPs [19]) present in HMWF and released once the proteins are denatured.

**Fig 4:**
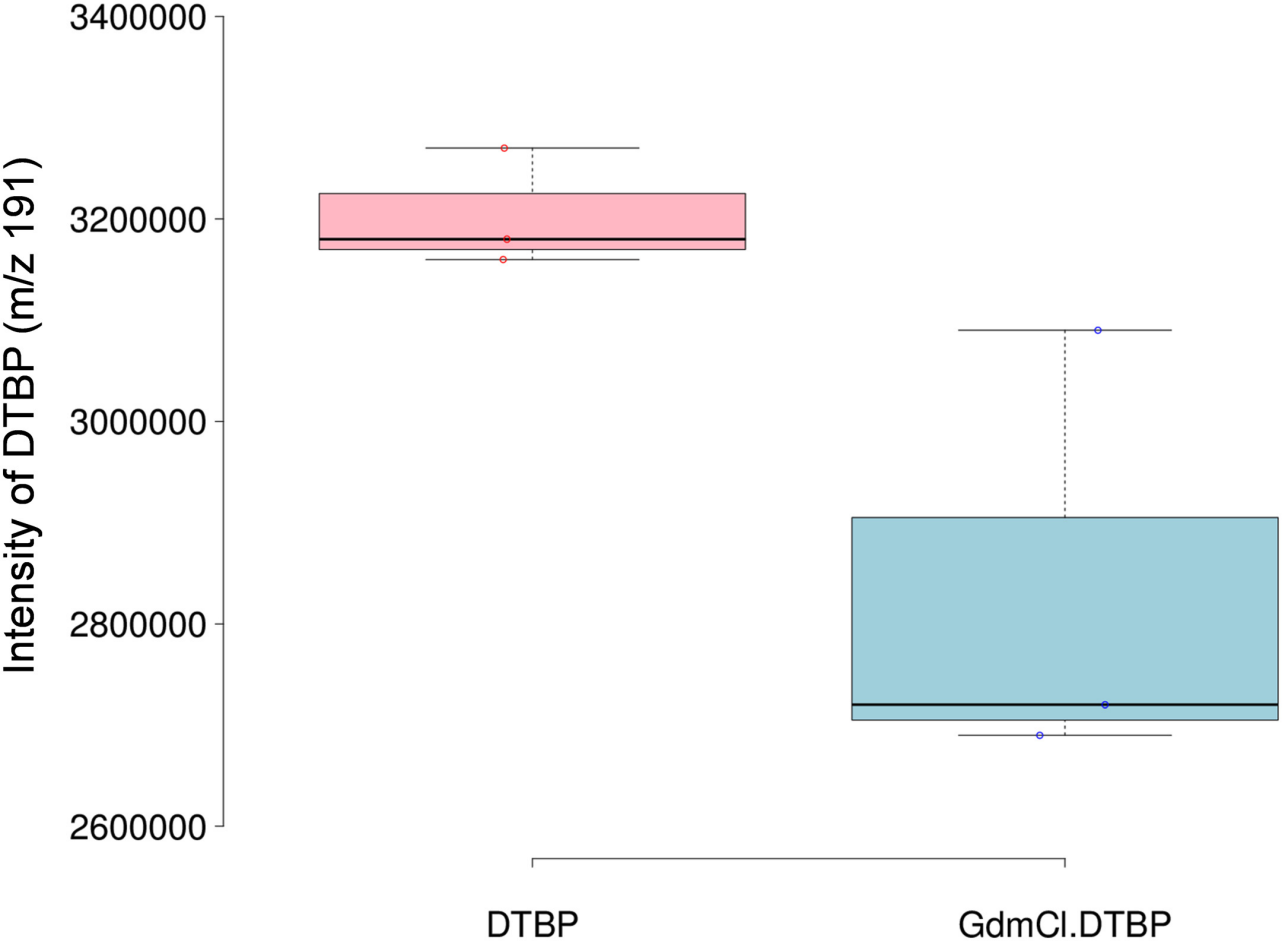
Changes in the release of DTBP upon addition of GdmCl to a DTBP solution in water. The box plot was created online using the BoxPlotR application [18]; http://boxplot.tyerslab.com/.

To further test the toxic waste hypothesis, two experiments were conducted. First, to investigate whether the increased release of DTBP in mouse urine upon addition of GdmCl is due to a decrease in the solubility of organic volatile molecules in urine and their consequent release into the headspace (a “salting out” effect), a DTBP solution was placed to a 4 mL glass vial and the headspace was analyzed in the presence or absence of GdmCl. As shown in Fig 4, the addition of GdmCl did not release DTBP, and rather decreased the release, suggesting that the increased release of DTBP in mouse urine upon addition of GdmCl did not result from a salting out effect from the protein denaturant. Second, we investigated whether SBT in the HMWF isolated from male mouse urine is displaced by the addition of DTBP to the fraction. If DTBP is a MUP ligand, it would bind to MUPs and subsequently displace SBT that had been bound to MUPs as previously demonstrated with other ligands such as menadione and BHT [6, 9]. The displacement of SBT was determined by the degree of SBT release after addition of DTBP. A substantially increased release of SBT was observed upon addition of DTBP, but not as pronounced as the release due to denaturation of MUPs (Fig 3C). This result provides further evidence that DTBP is a MUP ligand.

## Discussion

We unexpectedly detected DTBP, an exogenous compound and a potential toxin, in the urine of male and female wild-derived house mice living in standard colony conditions and in semi-natural enclosures. Whether DTBP causes harmful effects in mice remains unclear. DTBP was reported to be nontoxic to mother rats fed a diet containing DTBP for 21 days; however, the number of implantations and litters decreased [20]. We aimed to determine the source of exposure to DTBP, and our results show that exposure was not limited to our standard colony, indicating there was a source of contamination these mice share in common (i.e., food, water, or bedding). Indeed, DTBP was detected in the food (Data not shown).

Our results in this study suggest that DTBP is a MUP ligand, as MUPs are the major proteins detected in male and female mouse urine and bind a variety of ligands [6, 17, 21, 22]. Denaturation of MUPs by addition of GdmCl increased the release of DTBP, and this result was not due to the “salting out” effect (Fig 4). Thus, it is plausible that DTBP had been previously bound to MUPs and then released upon denaturation. Furthermore, when DTBP was added to the HMWF obtained from male urine, a substantially increased release of SBT, the major ligand of MUPs, was observed (Fig 3C), indicating that DTBP was bound to MUPs and displaced SBT. The increased release of DTBP from urine upon denaturation of MUPs (Fig 2A) and the fact that DTBP is structurally closely related to BHT, a previously identified MUP ligand [6], strongly suggest that DTBP is a MUP ligand.

MUPs may function to eliminate toxic wastes, as well as transport pheromones, and although these are not mutually exclusive hypotheses, there could be functional tradeoffs. Each MUP molecule binds a single ligand, and therefore, MUPs are expected to show competitive ligand binding [23]. We found that DTBP displaces SBT, a male-specific pheromone, in male urine (Fig 3C), which is consistent with previous findings that exogenous ligands, such as menadione and BHT, can displace MUP-bound SBT molecules [6, 9]. These findings suggest that there may be competitive binding between pheromone and toxin ligands for MUP binding pockets, at least in male mice (as there are no known MUP-dependent pheromones in female mice, this tradeoff may only apply to males). Thus, male mice may face a tradeoff between producing MUPs needed to eliminate toxic wastes versus transporting pheromone ligands. Males might escape this tradeoff by producing more MUPs overall or by regulating the expression of certain MUPs, such as the male-specific MUP (MUP20 ‘Darcin’ [24]), which has a high binding affinity for SBT [25]. Future studies are needed to investigate competitive ligand binding, and the possible tradeoffs between toxic waste elimination versus pheromone transport.

In summary, our results provide evidence that DTBP, an exogenous toxin, is bound to MUPs, which supports the hypothesis that MUPs function to bind and eliminate toxic waste (‘toxic waste hypothesis’). Our experiments do not rule out the possibility that DTBP is bound by another carrier protein in the urine, and therefore more experiments are needed to confirm our findings. Future studies are also needed to address how harmful xenobiotics are recognized and whether MUPs bind to most or only some toxins. It would be particularly interesting to determine whether toxins show competitive ligand binding with pheromones, and whether MUPs show different binding affinities for toxins and pheromones. Finally, if MUPs provide a toxic waste disposal system, studies need to investigate whether this mechanism increases survival and thus helps explain the evolution of *Mup* genes and MUP expression in different species [26, 27]. Previous functional studies on MUPs have focused on chemical signaling, especially the barcode hypothesis [2], but other hypotheses need to be investigated to explain why MUP production and profiles are variable, complex and dynamic [28].

## References

[1] Flower DR. The lipocalin protein family: structure and function. Biochem J. 1996;318(1):1–14. 10.1042/bj3180001 8761444 PMC1217580

[2] Beynon RJ, Hurst JL. Multiple roles of major urinary proteins in the house mouse, *Mus domesticus*. Biochem Soc Trans. 2003;31(1):142–6. 10.1042/bst0310142 12546672

[3] Hurst JL, Robertson DHL, Tolladay U, Beynon RJ. Proteins in urine scent marks of male house mice extend the longevity of olfactory signals. Anim Behav. 1998;55(5):1289–97. 10.1006/anbe.1997.0650 9632512

[4] Timm DE, Baker LJ, Mueller H, Zidek L, Novotny MV. Structural basis of pheromone binding to mouse major urinary protein (MUP-I). Protein Sci. 2001;10(5):997–1004. 10.1110/ps.52201 11316880 PMC2374202

[5] Stopková R, Hladovcová D, Kokavec J, Vyoral D, Stopka P. Multiple roles of secretory lipocalins (Mup, Obp) in mice. Folia Zool. 2009;58(Suppl. 1):29–40.

[6] Kwak J, Josue J, Faranda A, Opiekun MC, Preti G, Osada K, et al Butylated Hydroxytoluene Is a Ligand of Urinary Proteins Derived from Female Mice. Chem Senses. 2011;36(5):443–52. 10.1093/chemse/bjr015 21398415

[7] Stopková R, Dudková B, Hájková P, Stopka P. Complementary roles of mouse lipocalins in chemical communication and immunity. Biochem Soc Trans. 2014;42:893–8. 10.1042/bst20140053 25109975

[8] Larsen GL, Bergman Å, Klasson-wehler E. A methylsulphonyl metabolite of a polychlorinated biphenyl can serve as a ligand for α2μ-globulin in rat and major-urinary-protein in mice. Xenobiotica. 1990;20(12):1343–52. 10.3109/00498259009046632 1706122

[9] Robertson D, Hurst J, Hubbard S, Gaskell SJ, Beynon R. Ligands of urinary lipocalins from the mouse: Uptake of environmentally derived chemicals. J Chem Ecol. 1998;24(7):1127–40. 10.1023/a:1022434300449

[10] Hakk H, Diliberto JJ, Birnbaum LS. The effect of dose on 2,3,7,8-TCDD tissue distribution, metabolism and elimination in CYP1A2 (-/-) knockout and C57BL/6N parental strains of mice. Toxicology and Applied Pharmacology. 2009;241(1):119–26. 10.1016/j.taap.2009.08.009 19695277

[11] Staskal DF, Diliberto JJ, DeVito MJ, Birnbaum LS. Toxicokinetics of BDE 47 in Female Mice: Effect of Dose, Route of Exposure, and Time. Toxicological Sciences. 2005;83(2):215–23. 10.1093/toxsci/kfi018 15509665

[12] Staskal DF, Hakk H, Bauer D, Diliberto JJ, Birnbaum LS. Toxicokinetics of Polybrominated Diphenyl Ether Congeners 47, 99, 100, and 153 in Mice. Toxicological Sciences. 2006;94(1):28–37. 10.1093/toxsci/kfl091 16936226

[13] Choi SJ, Kim JK, Kim HK, Harris K, Kim C-J, Park GG, et al 2, 4-Di-tert-butylphenol from sweet potato protects against oxidative stress in PC12 cells and in mice. Journal of medicinal food. 2013;16(11):977–83. 10.1089/jmf.2012.2739 24074359 PMC3833388

[14] Yoon M-A, Jeong T-S, Park D-S, Xu M-Z, Oh H-W, Song K-B, et al Antioxidant effects of quinoline alkaloids and 2, 4-di-tert-butylphenol isolated from Scolopendra subspinipes. Biological and Pharmaceutical Bulletin. 2006;29(4):735–9. 16595909 10.1248/bpb.29.735

[15] Product Safety Summary Phenol, 2,4-bis-(1,1-dimethylethyl)- [cited 2016 10 Feb]. Available from: http://www.siigroup.com/EHSPdf/24-DTBPGPS.pdf.

[16] Kwak J, Grigsby CC, Preti G, Rizki MM, Yamazaki K, Beauchamp GK. Changes in volatile compounds of mouse urine as it ages: Their interactions with water and urinary proteins. Physiol Behav. 2013;120:211–9. 10.1016/j.physbeh.2013.08.011 23958471

[17] Kwak J, Grigsby CC, Rizki MM, Preti G, Köksal M, Josue J, et al Differential binding between volatile ligands and major urinary proteins due to genetic variation in mice. Physiol Behav. 2012;107(1):112–20. 10.1016/j.physbeh.2012.06.008 22728785

[18] Spitzer M, Wildenhain J, Rappsilber J, Tyers M. BoxPlotR: a web tool for generation of box plots. Nature Methods. 2014;11(2):121–2. 10.1038/nmeth.2811 24481215 PMC3930876

[19] Hurst JL, Beynon RJ. Scent wars: the chemobiology of competitive signalling in mice. BioEssays. 2004;26(12):1288–98. 10.1002/bies.20147 15551272

[20] Telford IR, Woodruff CS, Linford RH. Fetal resorption in the rat as influenced by certain antioxidants. Am J Anat. 1962;110(1):29–36. 10.1002/aja.1001100104 13920140

[21] Novotny MV, Ma W, Wiesler D, Žídek L. Positive identification of the puberty-accelerating pheromone of the house mouse: the volatile ligands associating with the major urinary protein. Proc R Soc Lond B. 1999;266(1432):2017–22. 10.1098/rspb.1999.0880 PMC1690305 10584338

[22] Bacchini A, Gaetani E, Cavaggioni A. Pheromone binding proteins of the mouse, *Mus musculus*. Experientia. 1992;48(4):419–21. 10.1007/BF01923448 1374722

[23] Sharrow SD, Vaughn JL, Žídek L, Novotny MV, Stone MJ. Pheromone binding by polymorphic mouse major urinary proteins. Protein Sci. 2002;11:2247–56. 10.1110/ps.0204202 12192080 PMC2373590

[24] Roberts SA, Simpson DM, Armstrong SD, Davidson AJ, Robertson DH, McLean L, et al Darcin: a male pheromone that stimulates female memory and sexual attraction to an individual male's odour. BMC Biol. 2010;8:21 10.1186/1741-7007-8-75 20525243 PMC2890510

[25] Phelan MM, McLean L, Armstrong SD, Hurst JL, Beynon RJ, Lian LY. The Structure, Stability and Pheromone Binding of the Male Mouse Protein Sex Pheromone Darcin. PLoS ONE. 2014;9(10):e108415 10.1371/journal.pone.0108415 25279835 PMC4184797

[26] Logan DW, Marton TF, Stowers L. Species Specificity in Major Urinary Proteins by Parallel Evolution. PLoS ONE. 2008;3(9):e3280 10.1371/journal.pone.0003280 18815613 PMC2533699

[27] Hagemeyer P, Begall S, Janotova K, Todrank J, Heth G, Jedelsky PL, et al Searching for Major Urinary Proteins (MUPs) as Chemosignals in Urine of Subterranean Rodents. J Chem Ecol. 2011;37:687–94. 10.1007/s10886-011-9971-y 21647723

[28] Thoß M, Luzynski K, Ante M, Miller I, Penn DJ. Major urinary protein (MUP) profiles show dynamic changes rather than individual "barcode" signatures. Front Ecol Evol. 2015;3:71 10.3389/fevo.2015.00071 26973837 PMC4783862

